# Characterization of Post COVID‑19 pneumo‑hematocele in the largest COVID-19 center in Mexico City

**DOI:** 10.1186/s13019-026-04218-2

**Published:** 2026-04-25

**Authors:** Guillermo Edmundo Castrillo-Hernández, Adriana Simoneta Pimienta-Ibarra, Edwin Gustavo Barrientos-Morales, Andrea Gloria Solares-Espinoza, Vanessa Dávila-Conn, Carmen Margarita Hérnandez-Cárdenas, Armando Roberto Castorena-Maldonado, Francina Valezka Bolaños, Alma Lilia Juárez-Armenta

**Affiliations:** National Institute of Respiratory Diseases (Mexico), INER, Calz. de Tlalpan 4502, Belisario Domínguez Secc 16, Tlalpan, 14080 Mexico City, Mexico

**Keywords:** COVID-19, Video-assisted thoracoscopic surgery (VATS), Pneumo-hematocele, Minimally invasive surgery, Postoperative air leak, Pneumothorax, Complications

## Abstract

**Background:**

Surgical management of post-COVID pneumo-hematocele is uncommon, and evidence regarding outcomes and risk factors for complications following video-assisted thoracoscopic surgery (VATS) is limited.

**Objective:**

To describe the clinical and surgical outcomes of video-assisted thoracoscopic surgery (VATS) for pneumo-hematocele in patients treated at the largest COVID-19 tertiary care center in Mexico City, and to explore associations between lesion characteristics and postoperative complications.

**Methods:**

We conducted a retrospective analysis of consecutive hospitalized COVID-19 patients with pneumo-hematocele who underwent VATS between September 2020 and September 2021. Sociodemographic, clinical, radiological, and surgical variables were collected. Exploratory logistic regression analyses with bootstrap validation and Firth penalization were performed to assess associations between lesion size and postoperative complications. A ROC curve analysis was used to identify an exploratory size threshold.

**Results:**

Eighteen male patients were included, with a median age of 37 years (32.8–50.8); 61.1% were active smokers, and 27.8% had a history of mechanical ventilation. Most patients (77.8%) were symptomatic prior to diagnosis, most commonly with dyspnea. Lesions were predominantly unilateral (66.7%) and most frequently involved the right lower lobe. Pneumothorax occurred in 88.9% of cases and was managed preoperatively with pleural drainage. No patient required surgical reintervention. Postoperative air leakage occurred in 33.3% of patients but resolved in less than 72 h in all cases. In exploratory analyses, a lesion diameter ≥ 8.3 mm was associated with increased risk of postoperative air leakage (OR 13.33, 95% CI 1.27–54.00, *p* = 0.044), with a sensitivity of 83.3% and specificity of 72.7%. The median hospital stay was 4 days (3.0–5.8), and no surgery-related deaths were observed.

**Conclusions:**

Video-assisted thoracoscopic surgery (VATS) is a safe surgical option for the management of post-COVID-19 pneumo-hematocele. In this exploratory analysis, larger lesion diameter (≥ 8.3 mm) was associated with an increased risk of postoperative air leakage. If validated in larger cohorts, this threshold could help identify patients who may benefit from closer postoperative monitoring or preventive intraoperative measures such as reinforced stapling techniques.

## Introduction

In 2019, the novel coronavirus SARS-CoV-2 was identified as the cause of COVID-19, an acute respiratory syndrome marked by hyperinflammation and a wide clinical spectrum [1, 2]. Disease outcomes have evolved due to vaccination, viral variants, and public health measures [2]. Nonetheless, 5–12% of patients require ICU admission and prolonged mechanical ventilation, which—along with COVID-19 pathology itself—can lead to complications requiring surgery [3]. Among these, pneumatocele and pneumo-hematocele have emerged as post-COVID sequelae affecting long-term health and quality of life [4]. A pneumatocele is a gas-filled cavity; when filled with blood, it is termed pneumo-hematocele [5]. COVID-19 patients are at higher risk of barotrauma than those with ARDS from other causes, increasing the likelihood of such lesions [6]. While often asymptomatic, complications such as pneumothorax may arise, prompting imaging-based diagnosis and, in some cases, surgical resection [5, 7]. Treatment options include observation, drainage, fibrin sealants, or video-assisted thoracoscopic surgery (VATS), with VATS preferred in surgical cases [8]. The National Institute of Respiratory Diseases (INER) in Mexico City became the country’s largest COVID-19 ICU center during the pandemic, significantly increasing mechanical ventilation cases [9–11]. Post-COVID pneumo-hematocele requiring surgical intervention remains poorly characterized, and predictors of postoperative complications are unknown. We aimed to identify risk factors for adverse outcomes following video-assisted thoracoscopic surgery (VATS) in patients with COVID-19-related pneumo-hematocele at Latin America’s largest COVID-19 referral center, informing best practices and reducing long-term healthcare burdens [10].

## Subjects

Between September 2020 and September 2021, INER admitted approximately 4,000 patients with confirmed SARS-CoV-2 infection. A total of 18 patients (0.45%) developed pneumo-hematocele meeting surgical criteria and underwent video-assisted thoracoscopic surgery (VATS), representing the complete surgical experience during this period. Patients with incomplete medical records or absolute contraindications for surgery were excluded.

## Methods

Sociodemographic, clinical, radiological, and surgical variables were extracted from electronic medical records, including age, sex, smoking history, mechanical ventilation history, lesion characteristics (location, size, laterality), and postoperative outcomes. Pneumo-hematocele was diagnosed based on CT imaging in all 18 patients. Surgical resection was indicated in those presenting with symptoms such as chest pain, dyspnea, or pleural effusion with findings consistent with pneumatocele or lung abscess. Operative decisions followed consistent clinical criteria. Patients underwent VATS in lateral decubitus position according to the affected side. Port placement was tailored to lesion location, with the number of ports determined by the treating thoracic surgeon. The approach was made at the 4th or 5th intercostal space. Following thoracoscopy and assessment, non-anatomical resections were performed, preserving maximal healthy tissue. Reinforced stapling was used selectively in one case based on intraoperative assessment of parenchymal friability; standard lung parenchymal staplers were employed in the remaining cases. Pleural drainage was placed using Argyle or Blake tubes connected to negative suction set at − 20 cm H₂O. Patients were discharged upon achieving clinical stability and adequate lung expansion.

Descriptive statistics included median and interquartile range [IQR] for continuous variables, and frequencies and percentages for categorical variables. Comparisons between unilateral and bilateral pneumo-hematocele were performed using Fisher’s exact test for categorical variables and Mann-Whitney U test for continuous variables, with *p* ≤ 0.05 considered statistically significant. Logistic regression analysis evaluated the association between lesion diameter and postoperative air leakage. An exploratory diameter cutoff was identified using ROC curve analysis with the Youden index. Given the small sample size, three complementary approaches were employed to ensure estimation stability and reduce small-sample bias: classical generalized linear models GLM, bootstrap resampling (1,000 iterations), and Firth’s penalized logistic regression. The study was approved by the hospital’s ethics committee.

## Results

All 18 patients were male, with a median age of 37 years (IQR 32.8–50.8). Eleven patients (61.1%) had a history of active smoking, with a median smoking index of 2.7 (2.0–8.9) pack-years. Five patients (27.8%) had required mechanical ventilation during acute COVID-19 illness. Most patients (88.9%) reported symptoms before pneumo-hematocele diagnosis, with dyspnea being the most common (77.8%). The median time from SARS-CoV-2 diagnosis to pneumo-hematocele diagnosis was 32.0 days (23.0–42.8). Sixteen patients (88.9%) developed associated pneumothorax, which required pleural drainage in the emergency department prior to surgical intervention (Table 1).

**Table 1:** Demographic characteristics and history of SARS-CoV-2 infection in patients with pneumo-hematocele

	Bilateral (*N* = 6)	Unilateral (*N* = 12)	Overall (*N* = 18)
Age (years)	44.5 [34.2, 51.0]	36.0 [34.2, 48.2]	37.0 [32.8, 50.8]
Assigned sex at birth (male)	6 (100%)	12 (100%)	18 (100%)
Active smoking	4 (66.7%)	7 (58.3%)	11 (61.1%)
Smoking Index (score)	2.4 [1.9, 5.8]	2.7 [2.0, 8.8]	2.7 [2.0, 8.9]
Systemic arterial hypertension	2 (33.3%)	0 (0%)	2 (11.1%)
Hospitalized due to COVID-19	1 (16.7%)	4 (33.3%)	5 (27.8%)
History of mechanical ventilation	0 (0%)	2 (16.7%)	2 (11.1%)
Pneumothorax	6 (100%)	10 (83.3%)	16 (88.9%)
Time from SARS-CoV-2 to P-H diagnosis (days)	23.0 [18.5, 25.2]	36.0 [30.5, 45.8]	32.0 [23.0, 42.8]
Symptomatic	1 (16.7%)	1 (8.3%)	2 (11.1%)
Cough	5 (83.3%)	5 (41.7%)	10 (55.6%)
Pleuritic pain	0 (0%)	4 (33.3%)	4 (22.2%)
Hemoptysis	1 (16.7%)	3 (25.0%)	4 (22.2%)
Dyspnea	6 (100%)	8 (66.7%)	14 (77.8%)
Postoperative Air Leakage	0 (0%)	6 (50.0%)	6 (33.3%)
Postoperative Complications	2.0 [2.0, 2.0]	2.0 [2.0, 2.0]	2.0 [2.0, 2.0]

P-H: pneumo-hematocele

CT imaging was performed in all cases, revealing unilateral lesions in 66.7% and bilateral in 33.3%. Lesions were peripheral in 83.3%, with the right lower lobe being the most commonly affected site (55.6%). A hydroaerial level was present in 94.4% of cases, and one case was initially interpreted as a pseudotumoral lesion (Table 2). Port placement varied according to lesion laterality. Among patients with unilateral pneumo-hematocele (*n* = 12), one port was used in 4 cases (33.3%) and two ports in 8 cases (66.7%). In patients with bilateral disease (*n* = 6), two ports were used in 3 cases (50.0%) and three ports in the remaining 3 cases (50.0%). No unilateral cases required three ports, and no bilateral cases were managed with a single port (*p* = 0.0275).

**Table 2:** Macroscopic characteristics of pneumo-hematocele

	Unilateral (*N* = 6)	Bilateral (*N* = 12)	Overall (*N* = 18)
Macroscopic aspect: *P*-H	6 (100%)	11 (91.7%)	17 (94.4%)
Tumor	0 (0%)	1 (8.3%)	1 (5.6%)
Central	1 (16.7%)	2 (16.7%)	3 (16.7%)
Peripheral	5 (83.3%)	10 (83.3%)	15 (83.3%)
Left lower lobe	1 (16.7%)	1 (8.3%)	2 (11.1%)
Left upper lobe	3 (50.0%)	2 (16.7%)	5 (27.8%)
Right lower lobe	3 (50.0%)	7 (58.3%)	10 (55.6%)
Right middle lobe	0 (0%)	1 (8.3%)	1 (5.6%)
Right upper lobe	2 (33.3%)	4 (33.3%)	6 (33.3%)
Largest diameter (mm)	8.0 [7.3, 8.8]	8.3 [7.2, 10.5]	8.1 [7.2, 10.0]

P-H: pneumo-hematocele

Preoperatively, all patients were classified as ASA II, and laboratory values were within normal ranges. All patients underwent VATS with non-anatomical resection. The median intraoperative blood loss was 100 ml (50.0–137.5). Postoperatively, 88.9% required pleural drainage, with Blake-type drains being used in 10 patients (55.6%) (Table 3).

**Table 3:** Characteristics of the surgical process and pre-surgical laboratory parameters in patients undergoing intervention for pneumo-hematocele

	Unilateral (*N* = 6)	Bilateral (*N* = 12)	Overall (*N* = 18)
Blood loss (mL)	125.0 [100.0, 150.0]	50.0 [50.0, 100.0]	100.0 [50.0, 137.5]
Pleural drainage	6 (100%)	10 (83.3%)	16 (88.9%)
Positive microbiological culture	5 (83.3%)	9 (75.0%)	14 (77.8%)
Hemoglobin (g/dL)	13.9 [13.8, 14.4]	14.6 [13.1, 15.9]	14.4 [13.2, 15.3]
Lymphocytes (×10³/µL)	1.4 [1.3, 1.5]	1.9 [1.2, 2.2]	1.5 [1.2, 1.9]
Leukocytes (×10³/µL)	8.5 [7.9, 9.8]	8.8 [6.8, 10.8]	8.5 [6.8, 10.5]
Neutrophils (×10³/µL)	6.7 [5.0, 7.4]	5.9 [3.5, 9.2]	6.3 [3.8, 7.6]
Platelets (×10³/µL)	245.0 [199.0, 416.0]	287.5 [209.2, 347.6]	286.0 [207.0, 352.6]
Neutrophil-to-lymphocyte ratio (NLR)	4.5 [3.0, 6.0]	3.8 [1.6, 5.0]	3.8 [2.2, 5.5]
Serum sodium (mEq/L)	137.0 [136.0, 139.0]	138.0 [137.0, 140.5]	138.0 [136.0, 140.0]

VATS: video-assisted thoracoscopic surgery; NLR: neutrophil to lymphocyte ratio; CRP: C-reactive protein

Postoperative air leakage occurred in 6 patients (33.3%), classified as forced expiratory leak in 4 (22.2%) and expiratory leak in 2 (11.1%); no inspiratory (continuous) air leaks were observed. All cases resolved with conservative management within 72 h. All cases were managed conservatively, and no patient required surgical reintervention. Most patients had no significant complications (Clavien-Dindo grade I, 14/18, 77.8%). One patient (5.6%) developed acute kidney injury managed medically without dialysis (Clavien-Dindo grade II). Two patients (11.1%) required ICU admission for reasons unrelated to the surgical procedure (Clavien-Dindo grade IV). The median hospital stay was 4 days (3.0–5.8). No surgery-related deaths occurred (Table 4).

**Table 4:** Postoperative events in patients undergoing surgery for pneumo-hematocele

	Overall (*N* = 18)
High-flow nasal cannula	1 (5.6%)
Mechanical ventilation	2 (11.1%)
Postoperative mortality (%)	0.0 (0%)
Vasopressors	2 (11.1%)
Air leak	6 (33.3%)
Surgical reintervention (%)	0.0 (0%)
Hospital stay (days)	4.0 [3.0, 5.8]
Clavien-Dindo grade	
— Grade I	14 (77.8%)
— Grade II	1 (5.6%)
— Grade IV	2 (11.1%)

ICU: intensive care unit; *up to 30 days after the surgery

Histopathological confirmation was obtained in 15 of 18 patients (83.3%). The most common findings were recent intraalveolar or subpleural hemorrhage (80.0%), subpleural bullae (66.7%), fibrinous pleuritis (53.3%), and organized pneumonia or pneumonic foci (46.7%). Additional findings included subpleural fibrosis (40.0%), vascular neoformation (26.7%), hemorrhagic infarcts (20.0%), and diffuse alveolar damage (6.7%). These features are consistent with the parenchymal injury pattern expected in post-COVID-19 pneumo-hematocele, reflecting a combination of barotrauma-related and inflammatory mechanisms.

To evaluate the relationship between lesion size and complications, exploratory logistic regression analyses were performed. Using the Youden index from the ROC curve, a lesion diameter ≥ 8.3 mm was identified as a potential cutoff threshold in this cohort. This cutoff was associated with postoperative air leakage (OR 13.33, 95% CI 1.27–54.00, *p* = 0.044), with a sensitivity of 83.3% and a specificity of 72.7%. Given the small sample size, three complementary analytical approaches were employed. Classical GLM yielded an OR of 13.33 (95% CI 1.41–321.63). Bootstrap resampling with 1,000 iterations produced a similar point estimate with a narrower confidence interval (OR 13.33, 95% CI 1.27–54.00). Firth’s penalized logistic regression yielded an OR of 8.91 (95% CI 1.18–115.07).

## Discussion

The predominance of male patients may reflect the higher severity of COVID-19 in men reported globally [12, 13]. The median age of 37 years is consistent with prior case reports of pneumatocele in younger COVID-19 patients [9, 14], which may be explained by increased chest wall compliance facilitating transmission of kinetic energy to lung parenchyma [10]. This age distribution may also relate to Mexico’s descending-age vaccination strategy during 2021, which left younger populations more vulnerable during the study period [15]. Risk factors such as male sex, younger age, and prior mechanical ventilation have been associated with pulmonary cystic lesions and barotrauma in COVID-19 [9]. In our series, 27.8% had required mechanical ventilation during acute illness. Pneumothorax occurred in 88.9% of cases and was managed preoperatively with pleural drainage, consistent with prior reports of pneumatocele rupture as a frequent complication [4]. Superinfection was documented in 22.2% of cases, which can evolve into empyema or parenchymal infection if not adequately managed [8]. No significant differences in clinical outcomes were observed between unilateral and bilateral pneumo-hematocele, reinforcing the feasibility and safety of VATS regardless of lesion laterality. Postoperative complications occurred in 33.3% of patients, predominantly air leakage, but all were managed conservatively without need for reoperation. The median hospital stay of 4 days and absence of surgery-related mortality support the safety profile of VATS for this indication [4, 8]. In our exploratory analysis, a lesion diameter ≥ 8.3 mm was associated with increased risk of postoperative air leakage (OR 13.33, *p* = 0.044). This finding was corroborated across three complementary analytical approaches, although validation in larger cohorts is needed. While our sample of 18 patients may appear limited, it represents the complete surgical cohort from Latin America’s largest COVID-19 referral center over 12 months (0.45% of 4,000 COVID-19 admissions), reflecting the true rarity of surgically managed pneumo-hematocele. To our knowledge, this is the largest single-center surgical series of COVID-19-related pneumo-hematocele reported to date. Conservative management remains first-line for uncomplicated cases; surgical intervention should be reserved for specific indications including persistent air leak, progressive growth, hemothorax, superinfection, or failure of conservative management [10], as applied in this study.

We acknowledge several limitations. The retrospective single-center design and absence of a control group preclude causal inference. The exclusively male cohort reflects the epidemiological pattern of severe COVID-19 at INER during the study period rather than a selection bias, though findings may not be generalizable to female patients or other healthcare settings. The small sample size limits statistical power and the precision of the estimated cutoff threshold, hence it should be considered exploratory.

## Conclusion

VATS is a safe and effective surgical option for post-COVID-19 pneumo-hematocele, with favorable short-term outcomes including no surgery-related mortality and no reoperations required. Although postoperative air leakage occurred in one-third of patients, all cases resolved with conservative management in less than 72 h, supporting the overall safety profile of this approach. In this exploratory analysis from the largest single-center surgical series reported to date, a lesion diameter ≥ 8.3 mm was associated with increased risk of postoperative air leakage. Prospective multicenter studies are warranted to establish evidence-based guidelines for the surgical management of post-COVID-19 pneumo-hematocele.

## Data Availability

The datasets generated and/or analysed during the current study are not publicly available due to patient privacy considerations but are available from the corresponding author on reasonable request.

## Abbreviations

Abbreviation: Definition
ARDS: Acute Respiratory Distress Syndrome
ASA: American Society of Anesthesiologists
CI: Confidence Interval
COVID-19: Coronavirus Disease 2019
CRP: C-Reactive Protein
CT: Computed Tomography
GLM: Generalized Linear Model
ICU: Intensive Care Unit
INER: Instituto Nacional de Enfermedades Respiratorias
IQR: Interquartile Range
NLR: Neutrophil-to-Lymphocyte Ratio
OR: Odds Ratio
ROC: Receiver Operating Characteristic
SARS-CoV-2: Severe Acute Respiratory Syndrome Coronavirus 2
VATS: Video-Assisted Thoracoscopic Surgery
